# Advancements in Minimally Invasive Techniques and Biomarkers for the Early Detection of Endometrial Cancer: A Comprehensive Review of Novel Diagnostic Approaches and Clinical Implications

**DOI:** 10.3390/jcm13247538

**Published:** 2024-12-11

**Authors:** Aleksandra Asaturova, Andrew Zaretsky, Aleksandra Rogozhina, Anna Tregubova, Alina Badlaeva

**Affiliations:** 1 Federal State Budgetary Institution “National Medical Research Center for Obstetrics, Gynecology and Perinatology Named after Academician V.I. Kulakov”, Ministry of Healthcare of the Russian Federation, Oparina Street, Bld. 4, 117997 Moscow, Russia; a-zaretsky@yandex.ru (A.Z.);; 2 Department of Molecular Technologies, Research Institute of Translational Medicine, N. I. Pirogov Russian National Research Medical University of the Ministry of Health of the Russian Federation, Bldg. 1, Ostrovityanova Street, 117997 Moscow, Russia

**Keywords:** endometrial cancer, early detection, liquid biopsy, DNA methylation, cervical cytology, non-invasive biomarkers, cancer screening, epigenetics, genetic biomarkers, cervicovaginal cytology

## Abstract

This review evaluates the advances in the early detection and diagnosis of endometrial cancer (EC), emphasizing the growing importance of minimally invasive techniques and novel biomarkers. Current diagnostic protocols for EC rely heavily on invasive procedures such as transvaginal ultrasound (TVU), hysteroscopy, and endometrial biopsy, which, although effective, can be overly burdensome for patients and inefficient for asymptomatic or low-risk populations. As there is no consensus on EC screening in high-risk or general populations, recent studies have explored alternative methods using biofluids and genomic biomarkers to improve sensitivity and specificity and facilitate access for patients. This review summarizes findings on DNA methylation markers, circulating tumor-derived nucleic acids, and the potential of liquid biopsy approaches for the early detection of EC. These innovations may not only streamline screening but also reduce the need for invasive procedures. This review highlights the potential of these biomarkers to be integrated seamlessly into the existing cervical cancer screening programs, which could transform screening methods for endometrial cancer and support the development of personalized, less invasive diagnostic procedures.

## 1. Introduction

Endometrial cancer (EC) is the most prevalent gynecological cancer and the fourth most common malignancy among women in developed countries, with 420,368 new cases reported in 2022 [1]. Unlike many other cancers, the incidence and mortality rates of endometrial cancer are rising globally [2,3], which is mainly due to the obesity epidemic [4,5]. However, when diagnosed at stages I or II, EC generally has a favorable prognosis [6]. The five-year survival rate for patients with stage I EC can reach up to 90%, while the rates drop to 50–60% in stage III and less than 20% in stage IV [7,8].

Timely diagnosis is crucial, as earlier detection significantly improves survival rates. Effective early detection of EC not only improves prognosis but also reduces the need for extensive surgery or adjuvant treatments, which ultimately lowers treatment costs and minimizes morbidity and mortality. This is particularly important for high-risk individuals, including those with obesity, metabolic syndrome, and Lynch syndrome [9,10].

Currently, there is no reliable screening tool to identify high-risk women suspected of having EC. Transvaginal ultrasonography (TVU) is the most commonly used technique and represents a valuable first step in the early diagnosis of EC thanks to its exceptionally high negative predictive value (99%). However, due to its relatively low specificity, additional tests are often required to rule out endometrial malignancy [11]. Inexpensive but uncomfortable endometrial biopsies frequently lead to false-negative results [12]. While endometrial biopsy is less stressful during hysteroscopy, vasovagal episodes and discomfort can contribute to hysteroscopy failure [13]. Therefore, there is an urgent need for precise and minimally invasive techniques for the early detection of EC.

**Figure 1:**
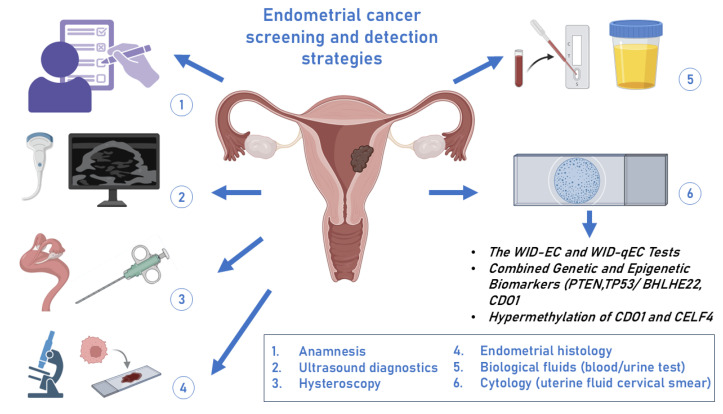
Current approaches to the detection and screening of endometrial cancer (created with Biorender platform). WID-EC—Women’s cancer risk IDentification-Endometrial Cancer, WID-qEC—Women’s cancer risk IDentification with Quantitative polymerase chain reaction test for Endometrial Cancer, *PTEN*—phosphatase and tensin homolog deleted on chromosome 10, *TP53*—Tumor protein P53, *BHLBE22*—Class E basic helix-loop-helix protein 22, *CD01*—Cysteine Dioxygenase Type 1, *CELF4*—CUGBP Elav-Like Family Member 4.

It is possible to obtain minimally invasive samples from peripheral blood, uterine lavage, cervicovaginal fluid, and other tumor-specific biofluids [14]. Gene sequencing of these biofluids can identify tumor-derived DNA, which could facilitate early tumor identification and subsequent early diagnosis of EC [15]. Current research is focused on developing less invasive approaches for the early detection of EC, particularly in conjunction with molecular testing. Significant progress has been made in this area in the past decades and we anticipate that less invasive methods will be effective for screening asymptomatic populations and improving current diagnostic protocols for high-risk and symptomatic women (Figure 1).

Abnormal postmenopausal bleeding (PMB) occurring more than one year after menopause, particularly when it presents as drip bleeding or bloody leucorrhea, is a major clinical feature of endometrial cancer (EC) or precancerous lesions [16]. Although screening strategies targeting women with PMB can detect up to 90% of malignant endometrial disease, only 5–10% of these women receive a diagnosis of malignant PMB rather than a diagnosis of malignant pathology [17].

Patients with perimenopausal and premenopausal EC may experience abnormal uterine bleeding (AUB) and menstrual cycle irregularities [18]. Nevertheless, it is important to note that 90% of patients with PMB do not have a malignant origin and 50% of women with PMB may have polyps, which can be easily detected and treated by hysteroscopy or endometrial biopsy [19].

Consequently, a thorough examination of the endometrium—including ultrasonography, endometrial biopsy (with or without hysteroscopy), and histopathologic evaluation—is essential to detect subtle pathology in all patients with these symptoms. The total cost of diagnostic evaluation of AUB and PMB is therefore substantial [20].

## 2. Transvaginal Ultrasound (TVU)

For patients at risk of endometrial cancer (EC), ultrasound examination—in particular transvaginal ultrasound (TVU)—is a well-tolerated and safe procedure. It can detect endometrial abnormalities, such as endometrial thickness, fluid in the uterine cavity, suspected polyps, or other abnormal features, all of which are associated with an increased risk of EC [21]. Numerous multicenter studies have confirmed that a TVU endometrial echo of ≤4 mm is sufficient for a preliminary assessment of postmenopausal bleeding (PMB), as the incidence of endometrial cancer drops to less than 1% when endometrial thickness is below this threshold [22].

However, due to the low specificity of TVU, women with a positive TVU test are likely to require further invasive tests and biopsies, which further increases the psychological and financial burden [23]. In addition, TVU is an operator-dependent test that requires a high level of expertise to obtain objective and reliable results. Factors such as an axial uterus, adenomyosis, coexisting fibroids, or previous surgical interventions can lead to unreliable TVU results [24].

Furthermore, physiological changes in sex hormones pose some challenges in assessing endometrial thickness (ET) in premenopausal and menopausal women [25]. The optimal ET threshold for these patients remains controversial [26]. Therefore, alternative, non-invasive triage techniques are required to assist the clinician in detecting the need for further investigation.

One such technique is saline infusion sonohysterography (SIS), which is another reliable method for assessing the endometrium [27]. Compared to TVU, SIS provides clearer images of the uterine cavity and increases the diagnostic accuracy for endometrial lesions, especially focal abnormalities [28].

It is important to note that type II EC, which is not estrogen-dependent and has a poorer prognosis, often develops independently of endometrial hyperplasia. In contrast, type I EC, which is associated with unopposed estrogen stimulation and a more favorable prognosis, is likely to occur together with a thickened endometrium and endometrial hyperplasia [29]. Studies have shown that 25–34% of patients with type II EC have a thin or unclear endometrial echo, suggesting that TVU may not be very useful for managing this subtype of cancer [30]. Therefore, if uterine bleeding is persistent or recurrent, endometrial examination is required regardless of the thickness of the endometrium.

## 3. Endometrial Biopsy and Histopathological Analysis

Endometrial biopsy followed by histopathological analysis is a frequently used method for diagnosing endometrial pathology. This can be performed via hysteroscopic endometrial biopsy, dilatation and curettage (D&C), or endometrial pipelle. Originally introduced by Cornier in 1984, the pipelle is an affordable and convenient device with minimal side effects, making it an attractive option for endometrial sampling [31]. However, pipelle sampling has a higher rate of sampling failure compared to D&C, often due to limited access to the uterine cavity or insufficient tissue collection [32]. Furthermore, studies have shown that pipelle biopsies are most effective for a homogenous endometrium and have limited sensitivity for detecting localized lesions [33].

As a result, D&C is still considered the gold standard for suspected cases [34], although it is more costly than pipelle sampling. The disadvantages of D&C, which include risks associated with anesthesia, infection, and uterine perforation, restrict its use to some extent [34,35]. Recently, hysteroscopy has become a valuable tool for endometrial biopsy as it offers a less invasive and precise method of examining the uterine cavity. Hysteroscopy allows for a direct visual assessment of suspicious lesions for biopsy or excision, which may reduce false-negative rates and enable a more accurate EC diagnosis [35,36]. Despite its advantages, hysteroscopy can be associated with procedural issues, particularly due to cervical stenosis or patient discomfort, which may hinder a complete examination [37].

Rare complications such as vasovagal reactions, local anesthetic toxicity, uterine perforation, fluid overload, and uterine bleeding can occur during hysteroscopy, although they are extremely rare. Preoperative administration of misoprostol, stabilization of loop power, and careful monitoring of the collected irrigating medium can help to minimize these risks [38]. Saline infusion sonohysterography (SIS)-guided endometrial aspiration is generally not a first-line method for endometrial biopsy as it does not improve diagnosis and carries potential risks of infection and tumor dissemination [12,39]. It is more suitable for cases where the diagnosis remains unclear after the initial biopsy or for patients in whom D&C or hysteroscopy is contraindicated [10,40].

Once adequate tissue has been collected, histopathological examination of the endometrium is performed to establish a definitive diagnosis. This provides valuable information on the classification of lesions as benign or malignant and thus helps to select the most appropriate therapeutic approach. Modern molecular tests can further support the accurate diagnosis of premalignant and malignant lesions [25].

Currently, the diagnostic procedure to confirm or exclude EC involves several sequential invasive tests. First, hysteroscopy is performed to evaluate the uterine cavity, followed by endometrial biopsy for histologic examination [41]. While most EC screening strategies to date have focused on symptomatic women, as they are more likely to have EC or precursor lesions, there is still considerable debate among gynecologists about the best approach for early EC detection in asymptomatic or high-risk populations [12]. An ideal screening method would be an accurate, cost-effective, and patient-friendly method that primarily aims to reassure low-risk individuals while identifying those at high risk for further invasive diagnostic procedures [42].

To summarize, invasive diagnostic procedures for endometrial disease, such as transvaginal ultrasound and endometrial biopsy, are associated with significant limitations. First, while transvaginal ultrasound is very sensitive, it often results in low specificity, making additional invasive procedures necessary. These procedures can increase the psychological and financial burden on patients and may require specialized training, which can make the results subjective. Second, endometrial biopsies, including those performed via pipelle or dilatation and curettage (D&C), can cause significant discomfort. Although minimally invasive hysteroscopy reduces some physical stress, complications such as vasovagal episodes, uterine perforation, and anesthetic reactions are still possible. Thirdly, pipelle biopsies, although cost-effective, can lead to false samples or inadequate tissue sampling, especially for focal lesions. Hysteroscopy allows for targeted tissue sampling but requires technical expertise and specialized equipment, further limiting its accessibility. Fourth, the variability in technique and interpretation, particularly in imaging and biopsies, underscores the difficulty in achieving consistent and reproducible diagnostic results. These points emphasize the need for the development of minimally invasive and patient-friendly diagnostic alternatives for the early and accurate detection of endometrial pathology. Such advances could overcome the limitations of current methods, improve patient compliance, and enable wider application in the clinical setting.

## 4. Non-Invasive Blood-Based Approaches for EC Detection

The development of minimally invasive sampling methods and the identification of cancer-specific biomarkers that can be detected in non-invasive “liquid biopsy” tests from various biofluids represent a significant advance in the diagnosis of EC. These methods are at the forefront of research because they are convenient, less invasive, and offer advantages in real-time sampling and repeatability. Such methods are a good alternative for patients who have difficulties with conventional biopsy [12]. While blood is a most important source for EC biomarkers, other body fluids, such as saliva, urine, cerebrospinal fluid, and even feces, have also shown potential as diagnostic media [42,43].

Blood-based molecular tests are known to support early cancer detection. However, due to the low concentration of biomarkers in serum, their effectiveness for EC is limited. Currently, no serum biomarker has achieved the sensitivity required for early diagnosis of EC, although HE4 has shown some promise [44]. Therefore, research has shifted towards analyzing circulating tumor-derived nucleic acids, such as genomic DNA, mitochondrial DNA, and short RNAs (mainly miRNAs) [45]. Studies show that patients with EC generally have higher levels of cell-free DNA (cfDNA) than patients with benign gynecologic conditions [46]. Furthermore, cfDNA levels tend to be elevated in high-grade EC [47].

Circulating tumor DNA (ctDNA) is a tumor-derived component of cfDNA that enters the bloodstream as a result of apoptosis, necrosis, or active release (exocytosis and shedding). Recent studies suggest that ctDNA is a promising tool for the early EC detection, with a 2022 study identifying aberrant methylation in genes such as *ZSCAN12* and *OXT* as potential markers for EC diagnosis [48]. At the same time, it should be noted that research on circulating tumor DNA in endometrial cancer is scant. Fiala et al. showed that the sensitivity of ctDNA detection in EC patients was below 10%, but the specificity reached 95% [49]. In addition, Dobrzycka et al. also reported that ctDNA was identified in the circulation of only 18% of individuals [50]. Consequently, this technology is rather inadequate as an early detection method for EC.

More promising results are likely to be related to the features of cfDNA fragmentomics features. In particular, Liu et al. found that the ensemble model had exceptional predictive accuracy and achieved high area under the curve (AUC) values in both training and independent validation cohorts. Its performance exceeded that of the individual base models, underlining the superiority of the ensemble approach. The established cut-off cancer scores exhibited high specificity and sensitivity, outperforming conventional methods such as transvaginal ultrasound in terms of specificity and circulating tumor DNA methods in terms of sensitivity. In particular, the classifier achieved a sensitivity of 96.4% in detecting early-stage (stage I) endometrial cancer and showed the potential to detect about 20% of cases at earlier stages, which could improve 5-year survival rates through improved diagnosis and intervention [51]. Despite the promising results, however, the study acknowledges certain limitations. The sample size was sufficient for an initial investigation but remains relatively modest and requires further validation in larger and more diverse clinical settings. To improve generalizability, measures were taken to mitigate batch effects, including an independent validation cohort. Future studies with a multi-site design are recommended to fully evaluate the performance of the model in different clinical settings.

MicroRNAs (miRNAs), endogenous non-coding RNA molecules involved in cellular processes such as tumor cell proliferation, differentiation and apoptosis, are also promising biomarkers for EC [52]. A recent meta-analysis has shown that certain circulating miRNAs, especially those identified from larger sample sizes and serum type clusters, have displayed high diagnostic accuracy with minimal publication bias. Certain miRNA signatures, including *miR-99a/miR-199b*, *miR-9/miR-1228/miR-92a*, *hsa-miR-200c-3p*, and *miR-222/miR-223/miR-186/miR-204*, have significant potential as non-invasive biomarkers for the early detection of EC [53,54]. In recent years, numerous publications describing in detail the individual components of this regulatory pathway, also known as the ‘axis’, have been published. This axis consists of a long non-coding RNA (lncRNA), a small non-coding RNA (competing endogenous RNA), and a target mRNA that interact with each other. In these pathways, mRNA and lncRNA compete for miR binding, and several linked pathways form what is now known as ceRNET (competing endogenous RNA network). The nodes in this network are ceRNAs, i.e., competing endogenous RNAs such as lncRNA and mRNA. The connections between them are defined by miRs [55,56].

Numerous ceRNA axes in endometrial cancer have been described in the literature, as have simpler interactions involving only two components on each proposed axis [57]. Recently, ceRNET was also mentioned by the European Commission. In 2019, Zhao and colleagues analyzed original data of EC RNA transcript data from The Cancer Genome Atlas (TCGA) database to identify prognostic biomarkers in EC [58]. Their study identified 62 lncRNAs, 26 miRs, and 70 mRNAs that are unregulated in EC. Ten lncRNAs, nineteen mRNAs, and four miRs showed a significant correlation with EC patients’ survival (*p* < 0.05). In 2022, Cai et al. investigated the TCGA and CPTAC databases to reconstruct an lncRNA-mediated ceRNA network for uterine corpus endometrial carcinoma [59]. The study made it possible to identify several axes, six of which have significant prognostic value. In the same year, Song et al. used the TGCA database to reconstruct a ceRNET comprising 5 deregulated lncRNAs, 7 deregulated miRs, and 90 deregulated mRNAs [60].

The identification of unregulated microRNAs in patients with EC is ongoing, and the identification of new competing endogenous RNA axes is relatively common. As an example, in a meta-analysis published in 2019, Delangle et al. [61] identified the dysregulation of 261 microRNAs (miRs) in EC, which were categorized into 133 onco-miRs, 110 onco-suppressor miRs, and 18 miRs with conflicting roles; of these, 139 showed dysregulation in endometrial tissue compared to benign and/or hyperplastic tissue [61].

However, the potential to use circulating miRs for this characterization has not yet been assessed and has been insufficiently investigated in EC. In 2022, Bloomfield and colleagues conducted a systematic review to identify circulating miRs in the serum and plasma of EC patients [62]. Their research found 33 highly deregulated miRs, of which 27 were upregulated and 4 were downregulated. The expression levels of the other two (*miR-21* and *miR-204*) varied from study to study. These studies suggest that appropriate combinations of miR expression levels may serve as prognostic markers that are useful in determining the histologic type and grade of EC, tumor size, FIGO stage, lymph node involvement, and survival, highlighting the role of miR in patient management decisions [61].

## 5. Pap Smear-Based Approaches for EC Detection (With a Special Focus on DNA Methylation Tests)

One of the most promising tools for the early diagnosis and prediction of endometrial cancer (EC) is the evaluation of Pap smears. Cervical smears are an ideal source of diagnostic material because cytology screening is widely available and there is a high participation rate of high-risk women in these programs. In addition, cervical cytology is non-invasive, requires no special equipment, and can be performed on an outpatient basis. Anatomically, endometrial cells can migrate directly toward the cervix, so early lesions can be detected in cervical smears before they progress to invasive carcinoma, provided the method is sufficiently sensitive.

The Papanicolaou (Pap) test, or cervical–vaginal cytology, has long been a proven method for the early detection of cervical cancer. In developed countries, organized public health efforts ensure that women regularly undergo a Pap test, which increases the moderate sensitivity of the test and contributes to a significant decrease in the incidence and mortality of cervical cancer. Because of the anatomical continuity between the uterine cavity, cervix, and vagina, the Pap test can also detect markers from the upper genital tract. In fact, George Papanicolaou, the inventor of the test, began studying the diagnostic potential of cervical and vaginal smears for the detection of EC towards the end of his career [63]. However, he found that cytology was less effective in detecting EC than it was for cervical cancer, as glandular abnormalities typical of endometrial adenocarcinoma are rarely identified in Pap smears, even in women aged 40 and above.

However, the molecular analysis of Pap smears may prove to be much more effective for the prediction or early detection of EC, as tests based on DNA, RNA, or proteins will capture not only whole cancer cells but also their fragments or metabolites, as well as molecular abnormalities shared by the tumor and its microenvironment (similar to the ‘field cancerization’ hypothesis), and even biomarkers of nearby cells’ reaction to tumor growth and local invasion.

A recent systematic review of 6599 cases of EC across 45 studies found that 45% of patients (95% CI, 40–50%) had abnormal Pap results prior to EC diagnosis or surgery, with no significant differences between conventional and liquid-based cytology methods. In particular, the percentage of abnormal results was significant in patients with aggressive histologies and advanced disease stages [64].

Advances in liquid-based technologies have made it possible to screen the same sample used for conventional HPV testing for genetic markers associated with other gynecologic cancers. Consequently, studies have investigated their potential to detect malignancies beyond cervical cancer and its precursors. Pap tests, as a minimally invasive sampling method, facilitate the detection of distant gynecologic cancers, such as ovarian and endometrial, mainly due to the enhanced sensitivity of molecular tests over morphologic examination in identifying early disease indicators [65]. Several proof-of-concept studies with limited sample sizes have shown that genomic, proteomic, or epigenomic analyses of Pap brush samples can distinguish between benign and malignant endometrial conditions [25].

Various testing methods for cervical smears have shown high specificity and sensitivity. In a notable study, Wang et al. used a highly sensitive NGS-based approach named Safe-SeqS to target 9392 unique nucleotide positions within 139 regions across 18 genes in a relatively large cohort (382 cancer patients and 714 controls) [66]. This test detected EC in 81% of patients (95% CI, 77–85%) using Pap brush samples, while sensitivity for detecting ovarian cancer samples was only 33% (95% CI, 27–39%). Reijnen et al. applied another NGS-based test to screen for mutations in high-risk genes, achieving 78% sensitivity and 97% specificity for EC detection [67]. In a similar study by Kinde et al., mutations in liquid Pap smear samples were identified in all EC cases, while none were detected in samples from women without cancer, achieving 100% sensitivity and specificity [68].

DNA methylation analysis remains one of the most promising approaches for cervical smear testing, as it has shown high accuracy, sensitivity, and specificity for multiple cancer types [68]. The methylation of CpG islands in gene promoter and enhancer regions offers a plethora of biomarkers for cancer screening, early detection, and monitoring. From a technological point of view, DNA methylation may be viewed as an ideal analyte, since it combines the versatility of RNA- and protein-based biomarkers with the stability of DNA. Cervical smears with DNA methylation assays have demonstrated considerable potential in identifying women at risk of cervical, endometrial, and ovarian cancer [69].

For example, Chang et al. and Huang et al. used quantitative methylation-specific polymerase chain reaction (qMS-PCR) and achieved a sensitivity and specificity of 83–92% and 69–95%, respectively, for EC detection [70,71]. Kim et al. also found that a similar approach provided 63% sensitivity and 96% specificity [72]. Heng et al. evaluated proprotein convertase (PC) activity via cleavage of a fluorogenic peptide substrate (FRET-KR-AMC) and observed significantly elevated PC activity in endocervical swabs from EC patients compared to controls [73]. Calis et al. assessed cancer antigen 125 (CA 125) levels using second-generation electro-chemiluminescent immunoassay and found a sensitivity of 78% and a specificity of 57% [74].

The WID-EC test (Women’s Cancer Risk Identification for Endometrial Cancer) was developed using cervical liquid-based cytology samples to measure DNA methylation at 500 CpG sites. Validation across multiple independent datasets demonstrated a high area under the curve (AUC) of 0.92 in the external validation sets and 0.82 in the prospective validation. The test showed strong diagnostic capabilities for EC detection, achieving 86% sensitivity and 90% specificity [69]. Concurrently, the WID-qEC test was developed and evaluated in a hospital-based cohort, using real-time PCR for DNA methylation of *ZSCAN12* and *GYPC* to identify endometrial and cervical malignancies. This test demonstrated excellent diagnostic accuracy, with an AUC of 0.99, a sensitivity of 100%, and a specificity of 82.5% for EC detection [75].

Researchers have also investigated mutations in genes such as *PTEN* and *TP53*, in combination with methylation markers like *BHLHE22* and *CDO1*. Although the specificity was relatively low, at 42%, the combination of these genetic abnormalities with methylation markers resulted in a sensitivity of 91.3%, increasing the sensitivity of detection without significant improvement in specificity [76]. The MPap test, another DNA methylation-based test, was evaluated for its ability to detect EC in women with abnormal uterine bleeding (AUB). This test outperformed transvaginal ultrasound (TVS) in sensitivity (92.5–92.9%) and specificity (71.7–73.8%) for EC detection, examining the methylation status of *BHLHE22* and *CDO1* in cervical smears [77].

The hypermethylation of the *CDO1* and *CELF4* genes has been shown to be a promising biomarker in cytologic samples. A dual methylation assay for these markers achieved a sensitivity of 84.9% and a specificity of 86.6% for the detection of atypical hyperplasia (AH) and EC in cervical cells. When combined with TVS to assess endometrial thickness, specificity increased to 94.9%, making this a non-invasive method for identifying women at high risk of developing EC [78].

In a separate study examining the methylation status of the *RASSF1A* and *HIST1H4F* genes, EC tissue samples showed significantly elevated levels of these methylation markers compared to normal controls. This test achieved an AUC of 0.938 for EC detection, suggesting that cervical smears may be a viable source of DNA for non-invasive EC diagnosis [79]. Furthermore, the potential for the detection of endometrial and ovarian cancer through cervical scrapings has been investigated, with promising results. High levels of methylation markers, including *PTGDR*, *HS3ST2*, *POU4F3*, and *MAGI2*, were observed in EC and ovarian cancer tissue. The study showed a sensitivity of 83–90% and a specificity of 69–75%, supporting the utility of methylation-based screening for clinical practice [70].

## 6. DNA Methylation Testing and Existing EC Screening Programs: Implementation Perspectives

The DNA methylation-based test is being actively discussed as a promising triage method for cervical cancer screening. This approach allows a more accurate risk assessment of precancerous lesions or invasive cancer, especially in women with a positive HPV result. The recently developed WID-qCIN test evaluates DNA methylation in three regions of human genes: *DPP6*, *RALYL*, and *GSX1* [80]. The study included women aged 30 years and older who participated in the screening program. When all 2377 HPV-positive samples were analyzed, the combination of WID-qCIN (with a predefined threshold) and HPV16/18 genotyping detected 93.4% of grade 3 CIN cases and 100% of invasive cervical cancers cases. This combination also predicted 69.4% of cases of CIN grade 2 or worse, compared to 18.2% identified by cytology. To detect one case of CIN grade 2 or worse over a six-year period, cytology-based triage required 4.1 referrals for colposcopy, while WID-qCIN/HPV16/18 required only 2.4 referrals. These results confirm that the combination of WID-qCIN and HPV16/18 is an improved triage strategy for HPV-positive women. The DNA methylation-based WID-qCIN test can complement HPV16/18 genotyping and offers improved enhanced performance compared to the widely used cytology. Notably, this approach does not rely on cellular morphology assessment and can be performed exclusively on DNA, making it suitable for screening strategies based on self-sampling. Implementing the WID-qCIN test alongside HPV16/18 screening could address the challenge of resampling patients for triage after a positive HPV result from self-collected samples.

Since cytological screening has been implemented in many countries and ensures high coverage, the introduction of an additional test system to analyze the same material could provide an additional advantage for minimally invasive diagnostic methods. This is particularly important as cervical smears can also be collected through self-sampling. Such an approach is not only cost-effective but also reduces the burden on healthcare systems [81].

As an alternative to cytology, testing objective biomarkers on minimally invasive samples has shown considerable potential and could be ideal for triaging patients with postmenopausal bleeding. DNA methylation in the promoter regions of tumor suppressor genes is a valuable biomarker for the detection of early-stage cancer. In the early stages of cancer development, promoter hypermethylation can lead to gene silencing and loss of tumor-suppressive function. Based on studies performed, special attention should be paid to the genes *ADCYAP1*, *BHLHE22*, *CDH13*, *CDO1*, *GALR1*, *GHSR*, *HAND2*, *SST*, and *ZIC1*, which have shown the best results in the detection of endometrial cancer in minimally invasive samples [82].

## 7. Emerging Alternative Molecular Methods for the Detection of EC

- Computer vision and deep learning: In the future, computer vision technologies and deep learning-based neural networks may be widely used to develop algorithms for the early detection of endometrial cancer using various imaging sources, including data obtained through ultrasound (US), magnetic resonance imaging (MRI), and hysteroscopy. These approaches have the potential to significantly improve diagnostic accuracy by comprehensively analyzing visual data and detecting subtle pathological changes. However, it should be noted that despite their advantages, these techniques methods and their analysis are not completely non-invasive tests, as they require diagnostic procedures involving a certain degree of intervention [83].
- Use of tampons for endometrial fluid collection: Studies suggest that the collection of fluid from the uterine cavity using tampons may be an effective method of detecting endometrial cancer. It was shown that the average duration of tampon use for fluid collection was 40 min. The goal is to develop an affordable at-home test that women can use independently. However, larger studies are needed to confirm the effectiveness of this method [84].
- Other biological markers in blood/urine/cervical smear/cervical fluid: Additional -omics-based approaches have yet to be investigated for early detection of endometrial cancer. Examples of these are discussed below.
  ◦ *Mass-spectrometry-based proteomics*: It was recently demonstrated that a five-marker panel of cervicovaginal fluid proteins including the immunoglobulin *mu* chain (IGHM), haptoglobin (HPT), fibrinogen alpha chain (FGA), lymphocyte antigen 6D (LY6D), and galectin-3-binding protein (LG3BP), as well as a three-marker panel of plasma proteins including HPT, proteasome 20S subunit alpha 7 (PSMA7), and apolipoprotein D (APOD), is capable of detecting EC with quite satisfactory sensitivity and specificity [85]. In addition, in a retrospective clinical study assessing 52 EC-related proteins in cervical fluid from 41 patients using targeted proteomics, Martinez-Garcia et al. identified SERPINH1, VIM, TAGLN, PPIA, CSE1L, and CTNNB1 as potential protein biomarkers for discrimination between EC and symptomatic non-EC women with abnormal uterine bleeding, achieving AUC > 0.8 [86].
  ◦ *Lipidomics*: A lipid biomarker panel, including ursodeoxycholic acid, PC(O-14:0_20:4), and Cer(d18:1/18:0), was established by Cheng et al. [87]. When evaluated as a diagnostic model to differentiate early-stage EC patients from healthy controls and patients with atypical endometrial hyperplasia, this model performed very well, with an AUC of up to 0.928. In particular, the study indicated that the lipid biomarker panel was superior to clinically established indicators for EC diagnosis, including HE-4, CA-125, CA-15.3, and CA-19.9, suggesting that it could be used as an excellent supplementary method for the diagnosis of early-stage EC. However, these results should be taken with a bit of caution due to the retrospective nature of the study and the limited number of samples analyzed.

Modern research emphasizes the great potential of new screening methods for endometrial cancer that aim at early and non-invasive detection. The use of computer vision and deep learning technologies enables the development of algorithms to analyze images obtained through ultrasound (US), magnetic resonance imaging (MRI), and hysteroscopy and can increase diagnostic accuracy by detecting minimal pathological changes. In addition, the development of methods for sampling endometrial fluid using tampons offers the possibility of developing an accessible at-home test that women can perform independently. Promising progress has also been made in the analysis of biomarkers in biological fluids, such as blood, urine, cervical smears, and fluids. Proteomics and lipidomics demonstrate high accuracy in identifying specific panels of proteins, lipids, and other molecules that can distinguish early-stage endometrial cancer from benign changes. These approaches have the potential to significantly improve early detection and screening but require further large-scale clinical studies to confirm their effectiveness in practice.

## 8. Clinical Implications and Future Directions

The application of DNA methylation biomarkers for the detection of endometrial cancer represents a promising alternative to conventional diagnostic methods and offers several key advantages:

- Non-invasiveness: Cervical smears provide a significantly less invasive approach compared to endometrial biopsies, making these tests more patient-friendly.
- High sensitivity and specificity: Many methylation-based tests have high sensitivity and specificity, which can help reduce false-positive rates and unnecessary invasive follow-up tests.
- Seamless integration into ongoing screening programs: These tests can often be performed alongside routine cervical cancer screening, allowing for an efficient utilization of existing healthcare infrastructure [69,77,79].

## 9. Limitations of This Review

Although this review shows promising advances in non-invasive biomarkers for the early detection of endometrial cancer (EC), several limitations should be noted. First, many of the studies cited are based on relatively small, heterogeneous cohorts, potentially limiting the generalizability of the results to broader populations. In addition, differences in methods, including differences in sample collection, processing, and analysis, lead to inconsistencies that make direct comparison between studies difficult. The variability of biomarkers and lack of standardization across clinical settings also pose challenges for clinical application, as results from one population may not translate well to other populations with different demographic or genetic backgrounds.

Although liquid biopsy techniques and DNA methylation markers show promise, their application in routine screening is still in the early stages. There are significant technical and economic barriers to widespread clinical application, as these advanced diagnostic methods often require specialized equipment and expertise that is not readily available in all healthcare facilities. Finally, certain biomarkers, such as circulating tumor DNA and microRNAs, are very sensitive. However, their specificity for early-stage EC needs to be further proven to reduce the risk of false-positive results, which could lead to patients having to undergo unnecessarily invasive follow-up tests, which would be stressful for them.

## 10. Conclusions

This review highlights the significant advances in non-invasive diagnostic methods for the early detection of endometrial cancer (EC), focusing on novel biomarkers and liquid biopsy techniques. Conventional diagnostic methods such as transvaginal ultrasound and endometrial biopsy, while effective, are often uncomfortable for patients and limited in early detection. New approaches using liquid biopsies—such as DNA methylation, circulating tumor DNA (ctDNA), and microRNA biomarkers—offer promising alternatives for high-risk populations. Studies show that specific methylation markers (e.g., *ZSCAN12*, *GYPC*, and *OXT*) and circulating miRNAs have high diagnostic accuracy in detecting early-stage EC, potentially allowing these techniques to be seamlessly integrated into routine cervical screening. These innovative biomarkers not only increase diagnostic sensitivity and specificity but also improve accessibility and convenience for patients by reducing the need for invasive procedures. In the future, these minimally invasive approaches could revolutionize cervical cancer screening and early diagnosis, ultimately contributing to a more efficient and individualized diagnosis for at-risk patients.

Future research should aim to further refine the specificity of these methylation tests to minimize false-positive results, especially in populations with benign uterine disease. In addition, longitudinal studies are essential to assess the predictive capacity of these biomarkers for cancer development in high-risk groups.
